# Psychological factors and biochemical indicators influencing sleep disturbance of patients with primary biliary cholangitis in China: a cross-sectional survey analysis

**DOI:** 10.3389/fmed.2024.1444473

**Published:** 2024-10-03

**Authors:** Chenyang Zhao, Bo Zang, Qixuan Liu, Bingqian Liu, Yuan Yao, Hua Li, Yifei Yang, Bin Liu

**Affiliations:** ^1^Department of Rheumatology, The Affiliated Hospital of Qingdao University, Qingdao, China; ^2^Boston University, Boston, MA, United States

**Keywords:** primary biliary cholangitis, sleep disturbance, Pittsburgh sleep quality index, generalized anxiety disorder scale, patient health questionnaire

## Abstract

**Objective:**

The impact of primary biliary cholangitis (PBC) on sleep disturbance is relevant to treatment decision-making processes. Studies on sleep disturbance in Chinese patients with PBC are still lacking.

**Methods:**

We analyzed and compared the health-related quality of life (HRQoL) of 107 PBC patients by using the Pittsburgh Sleep Quality Index (PSQI) questionnaire, Generalized Anxiety Disorder Scale (GAD-7), Patient Health Questionnaire 9 (PHQ-9), Short Form (36) Health Survey Questionnaire (SF-36), Fatigue Visual Analog Scale (F-VAS). Patients’ biochemical markers were also collected for correlation analysis with HRQoL. Receiver operating characteristic (ROC) curves and area under the curve (AUCs) were used to determine the diagnostic performance of PSQI, GAD-7, and biochemical markers for assessing the impaired liver function (Child–Pugh B–C) of PBC diagnosis.

**Results:**

Sixty-two (57.9%) PBC patients suffered from poor sleep quality (PSQI >5). The global PSQI score was positively correlated with GAD-7 (*r* = 0.561, *p* < 0.001), and PHQ-9 scores (*r* = 0.652, *p* < 0.001). There was a negative correlation (*r* = −0.216, *p* = 0.025) between sleep quality and red blood cell (RBC) count. PBC patients with poor sleep quality had significantly higher GAD-7 scores (5 vs. 0, *p* < 0.001), PHQ-9 scores (5.5 vs. 0, *p* < 0.001), and lower albumin levels (39.6 vs. 37.6 g/L, *p* = 0.040) than those with good sleep quality. Based on the SF-36 scores, PBC patients with poor sleep quality had lower physical functioning scores (85 vs. 80, *p* = 0.022), role physical scores (100 vs. 75, *p* = 0.007), and worse mental health (60 vs. 56, *p* = 0.002) than those with good sleep quality. ROC analyses showed that the AUC and optimal cut-off values of the combination of PSQI, GAD-7, and RBC for assessing the impaired liver function in PBC diagnosis were 0.771 and 0.193, respectively.

**Conclusion:**

The sleep disturbance was strongly correlated with the severity of anxiety, depression, and RBC count in PBC patients. Meanwhile, PBC patients with poor sleep had poor HRQoL and lower albumin levels. It is feasible to use the combination of PSQI, GAD-7, and RBC for initial screening of the impaired liver function in PBC. Besides routine blood biochemical and imaging indicators, evaluating mental health-related indicators in PBC patients is imperative.

## Introduction

The exact cause of PBC, a chronic liver disease characterized by cholestasis, is still unknown. Seventy years ago, the diagnosis of PBC was often associated with the stage of cirrhosis (1, 2). Early diagnosis of PBC has improved significantly with more accurate cholestasis measurement and anti-mitochondrial antibody (AMA) detection. In 2015, primary biliary cirrhosis was renamed as primary biliary cholangitis to describe the natural history of the disease more accurately and reduce the mental stress of PBC patients (3).

The prevalence of sleep disturbance among PBC patients reported in different articles varies, ranging from approximately 29 to 88%, due to ethnic, geographic, cultural, and social backgrounds (4–6). Generally, sleep regulates adaptive and innate immune responses by influencing the hypothalamus–pituitary–adrenal (HPA) axis and the sympathetic nervous system (SNS) (7). Sleep disturbance leads to activation of the HPA and SNS pathways, which contribute to an increased proinflammatory of the basal gene expression profile (8). Although a large number of studies have been conducted on the inflammatory cell pathways in PBC patients, the interplay between biochemical indicators, sleep, and psychological factors remains unknown, which needs to be explored. To measure the sleep and psychological factors of PBC patients, various non-invasive psychometric tools such as PSQI, GAD-7, PHQ-9, SF-36, and PBC-40 are widely used, and their reliabilities have been verified through practice (5, 9–11).

The purpose of the present study was to analyze the influencing factors of sleep disturbance and HRQoL in Chinese PBC patients, which were evaluated by PSQI, GAD-7, PHQ-9, and SF-36 and investigated their links with the severity of disease.

## Materials and methods

### Study cohort

Newly diagnosed PBC patients (aged ≥18 years) were hospitalized and prospectively enrolled between January 2021 and June 2023. The diagnosis of PBC was based on the 2018 Practice Guidance from the American Association for the Study of Liver Diseases (12). The exclusion criteria included patients who (a) exhibited other clearly diagnosed comorbid liver diseases, for instance, viral hepatitis, drug-related liver diseases, or fatty liver disease of alcoholic or non-alcoholic origin; (b) presented with obstructive cholestasis; and (c) were affected by other systemic diseases including autoimmune hepatitis, primary sclerosing cholangitis, and sarcoidosis. PBC patients received a standardized, self-completed questionnaire, and detailed biochemical indicators were collected during their visit to the Affiliated Hospital of Qingdao University. Only one staff member was responsible for collecting questionnaire answers and matching clinical information, and the patient’s personal information was anonymized in the subsequent analysis of the data. Written informed consent was obtained from all participants. The procedures followed in this study were approved by the Ethics Committee of the Affiliated Hospital of Qingdao University (approval no. QYFYWZLL 27020) and conformed to the ethical guidelines of the 1975 Declaration of Helsinki.

### Assessment of biochemical indicators

The laboratory investigation of red blood cell (RBC) count, white blood cell (WBC) count, and platelet (PLT) count was performed using automated hematology analyzers (Sangon Biotech Co., Ltd., Shanghai).

C-reactive protein (CRP), alanine aminotransferase (ALT), aspartate aminotransferase (AST), albumin(ALB), alkaline phosphatase (ALP), *γ*-glutamyl transpeptidase (γ-GT), total bilirubin (TBil), direct bilirubin (DBIL), and indirect bilirubin (IBIL) were measured using the automatic biochemical analyzer (Sangon Biotech Co., Ltd., Shanghai) and related reagents.

By following the manufacturer’s ELISA protocols (Sangon Biotech Co., Ltd., Shanghai), general laboratory testing for serological assays was performed, including assessing the values of immunoglobulin M (IgM), immunoglobulin G (IgG), immunoglobulin A (IgA), and immunoglobulin E (IgE).

Anti-nuclear antibody (ANA), anti-ribonucleoprotein (RNP), anti-Smith (anti-Sm), anti-Sjögren’s syndrome-related antigen A (SSA), anti-Sjögren’s syndrome-related antigen B (SSB), anti-double-stranded deoxyribonucleic acid (ds-DNA), anti-scleroderma-polymyositis (SCL-PM), anti-histidyl-tRNA synthetase (HRS = Jo-1), anti-centromere antibody (ACA), anti-proliferating cell nuclear antigen (PCNA), anti-nucleosome antibody (ANuA), anti-histone antibody (AHA), anti-ribosomal P protein antibody (anti-Rib-P), anti-mitochondrial antibody (AMA), M2 subtype of anti-mitochondrial antibody (AMA-M2) chemiluminescent immunoassay assay kits were obtained from HOB Biotech Group (BioCLIA Autoimmune, China), according to the manufacturer’s instructions described in the assay procedure. The assay was performed on the SMART 6500 instrument (Keysmile Biological Technology Co., Ltd., Chongqing).

### Assessment of sleep quality

The Chinese version of the PSQI was used to assess sleep quality and screen for sleep disturbance. It consisted of 19 items divided into 7 components: subjective sleep quality, sleep latency, sleep duration, habitual sleep efficiency, sleep disturbances, sleep medication use, and daytime dysfunction. The total score for the seven components was the global PSQI score, ranging from 0 to 21. Higher PSQI scores indicated poorer sleep quality. A global PSQI score of >5 indicated poor sleep quality (13).

### Assessment of anxiety and depressive symptoms

The Chinese versions of the GAD-7 and PHQ-9 were used to assess anxiety and depression, respectively, with scores ranging from 0 (none at all) to 3 (almost every day) over the past 2 weeks (14, 15). An overall GAD-7 or PHQ-9 score of ≥10 indicated the prevalence of anxiety symptoms and depressive symptoms. Any participant who scored above the therapeutic threshold (>10) on the PHQ-9 and GAD-7 was referred to the department of psychology for clinical evaluation.

### Assessment of HRQoL

HRQoL was measured by the Chinese version of SF-36, which was a widely used survey of self-reported physical and mental health with 36 questions that measure 9 dimensions of health: health transition (HT), physical functioning (PF), role physical (RP), bodily pain (BP), general health (GH), vitality (VT), social functioning (SF), role emotional (RE), and mental health (MH) (16). The aggregated scores for all dimensions range from 0 to 100, with higher scores indicating better HRQoL in PBC patients.

### Assessment of fatigue

F-VAS was typically comprised a 100-mm horizontal line, anchored by two statements representing extreme ends of a single fatigue continuum (“Not at all tired” to “Very tired”). Respondents were instructed to make a mark across or on the F-VAS line to describe the point between the two anchors that best-reflected fatigue status. A higher score on a scale of 0 to 10 represented a greater severity of fatigue. F-VAS greater than 2 was considered fatigue (17).

### Data analysis

The data were analyzed using SPSS v 25.0 (Chicago, IL, United States). The data were presented as the mean ± standard deviation (SD) and analyzed using implementing Student’s t-tests, Mann–Whitney U-tests, 2 × 2 χ^2^ tests, or Fisher’s exact assessments as appropriate based on the data type and distribution. The correlation coefficient between PSQI and HRQoL was determined using Spearman’s rank correlation test. ROC curves and AUCs were used to determine the diagnostic performance of the PSQI, GAD-7, RBC, and the combination of PSQI, GAD-7, and RBC in assessing the impaired liver function (Child–Pugh B–C) of PBC. The optimal cutoffs were determined based on the sensitivity and specificity. Normally distributed quantitative variables were expressed as mean ± SD, and non-normally distributed data were expressed as median (interquartile range). Descriptive statistics were presented as frequencies (n [%]). Statistical significance was defined at the 95% confidence level.

## Results

A total of 107 PBC patients were recruited, whose clinical information is shown in Table 1. There were 8 (7.4%) men and 99 (92.3%) women. Their ages ranged from 24 to 89 years old, with a median age of 62 years old.

**Table 1:** Results of the sleep state and serological assays for patients with PBC.

	Good sleep (*n* = 45)			Poor sleep (*n* = 62)			
	Q1	Q2	Q3	Q1	Q2	Q3	*p*
Age	53	63	71	50	57.5	68	0.131
RBC (10^12^/L)	3.43	3.89	4.30	3.18	3.78	4.10	0.196
WBC (10^9^/L)	2.98	4.42	5.59	4.12	4.94	5.83	0.171
PLT (10^9^/L)	109	172	203	83	162	222	0.920
CRP (mg/L)	0.50	1.34	6.57	0.50	1.83	6.30	0.477
ALB (g/L)	35.10	39.60	46.40	30.80	37.60	42.90	0.040
TBIL (umol/L)	11.50	16.40	21.10	10.70	16.30	32.40	0.553
DBIL (umol/L)	4.50	5.80	7.96	3.80	5.75	16.90	0.823
IBIL (umol/L)	6.90	8.70	13.70	6.60	9.00	16.00	0.382
(ALT/AST)	0.69	0.86	1.29	0.70	0.92	1.20	0.992
ALT (IU/L)	19.00	29.00	74.80	17.50	30.00	54.00	0.640
AST (IU/L)	19.10	34.00	65.50	21.00	33.65	59.90	0.900
ALP (IU/L)	73.00	101.00	251.50	65.70	103.50	189.00	0.745
γ-GT (IU/L)	28.40	65.00	180.00	27.80	78.80	176.00	0.887
IgM (g/L)	1.27	2.08	3.51	1.18	1.60	2.94	0.298
IgG (g/L)	13.80	15.40	21.90	11.40	14.75	20.20	0.116
IgA (g/L)	2.25	2.78	4.16	2.16	3.17	4.42	0.717
IgE (g/L)	13.29	19.00	84.01	17.70	25.82	70.00	0.367
Child–Pugh score	5	5	6	5	5.5	7	0.160

Data were median (interquartile range), proportions, or simple frequencies as appropriate. The first quartile (Q1) is the value under which 25% of data points are found when they are arranged in increasing order. The second quartile (Q2, or the median) is the 50th percentile, meaning that 50% of the data fall below the second quartile. The third quartile (Q3) is the value under which 75% of data points are found when arranged in increasing order. RBC, red blood cell; WBC, white blood cell; PLT, platelet count; CRP, C-reactive protein; ALT, alanine aminotransferase; AST, aspartate aminotransferase; ALB, albumin; ALP, alkaline phosphatase; d, cell; WBC, white blood cell; PLTBil, total bilirubin; DBIL, direct bilirubin; IBIL, indirect bilirubin; IgM, immunoglobulin M; IgG, immunoglobulin G; IgA, immunoglobulin A; IgE, immunoglobulin E.

### Sleep quality

**Figure 1:**
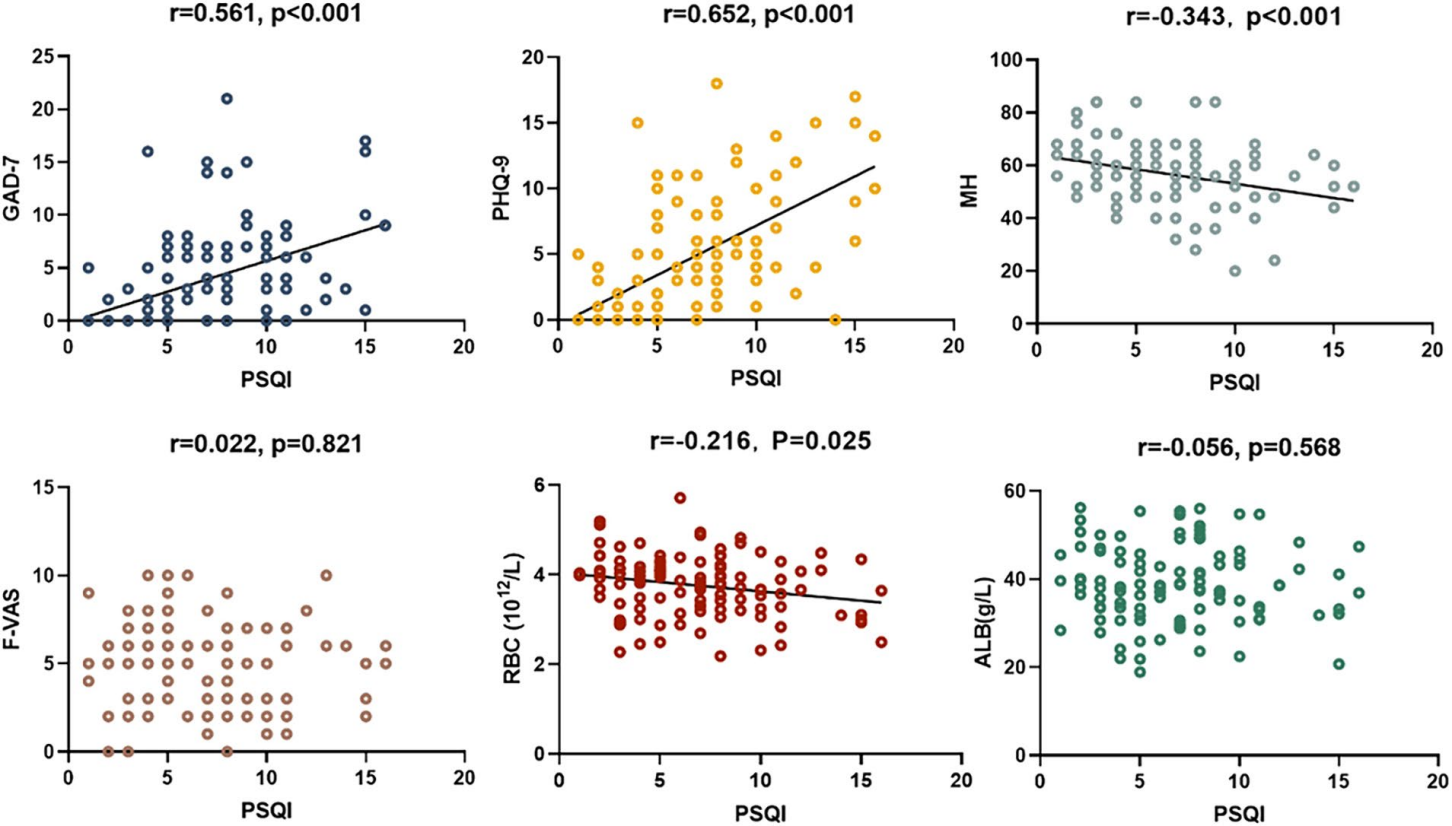
Correlation between PSQI, GAD-7, PHQ-9, MH, F-VAS, RBC, and ALB in patients with PBC.

Among the patients, 45 reported good sleep, while 62 reported poor sleep. It was observed that the patients with poor sleep quality and PBC had significantly lower albumin levels compared to those who reported good sleep (39.6 vs. 37.6 g/L, *p* = 0.040). However, there were no significant differences in other biochemical markers, including anti-ENA antibody profiles (Tables 1, 2). There was a negative correlation between sleep quality and RBC count (*r* = −0.216, *p* = 0.025; Figure 1). In addition, no correlation was found between sleep quality and other biochemical indicators (Table 3).

**Table 2:** Results of the sleep state and anti-ENA antibody profiles for patients with PBC.

	Good sleep (*n* = 45)		Poor sleep (*n* = 62)		*p*
Sex	3/42		5/57		1.000
ANA	44	97.78%	61	98.39%	1.000
Anti-RNP	2	4.44%	7	11.29%	0.298
Anti-sm	1	2.22%	3	4.84%	0.637
Anti-SSA	17	37.78%	26	41.94%	0.665
Anti-Ro-52	23	51.11%	30	48.39%	0.781
Anti-SSB	5	11.11%	11	17.74%	0.418
Anti-scl-70	0	0.00%	0	0.00%	
Anti-PM-Scl	1	2.22%	1	1.61%	1.000
Anti-Jo-1	0	0.00%	2	3.23%	0.508
ACA	16	35.56%	23	37.10%	0.870
Anti-PCNA	0	0.00%	2	3.23%	0.508
Anti-dsDNA	2	4.44%	6	9.68%	0.463
ANuA	1	2.22%	5	8.06%	0.397
AHA	1	2.22%	4	6.45%	0.395
anti-Rib-P	2	4.44%	1	1.61%	0.571
Anti GP210	10	22.22%	12	19.35%	0.717
Anti SP100	5	11.11%	6	9.68%	1.000
Positive AMA	34	75.56%	51	82.26%	0.397
Positive AMA-M2	31	68.89%	40	64.52%	0.637

Data were proportions or simple frequencies as appropriate. ANA, Anti-nuclear antibody; RNP, Ribonucleoprotein; anti-Sm, anti-Smith; SSA, anti-Sjögren’s syndrome-related antigen A; SSB, anti-Sjögren’s syndrome-related antigen B; ds-DNA, double-stranded deoxyribonucleic acid; SCL, scleroderma; PM, polymyositis; Jo-1, histidyl-tRNA synthetase (HRS = Jo-1); ACA, anti-centromere antibody; PCNA, proliferating cell nuclear antigen; ANuA, anti-nucleosome antibody; AHA, anti-histone antibody; anti-Rib-P, anti-ribosomal P protein antibody; AMA, anti-mitochondrial antibody; AMA-M2, M2 subtype of anti-mitochondrial antibody.

**Table 3:** Correlation between sleep state and age and serological assays for patients with PBC.

	*r*	*p*
Age	−0.095	0.331
RBC	−0.216	0.025
WBC	−0.176	0.069
PLT	−0.188	0.052
CRP	0.106	0.275
ALB	−0.056	0.568
TBIL	0.137	0.159
DBIL	0.165	0.089
IBIL	0.106	0.275
(ALT/AST)	0.173	0.075
ALT	0.015	0.876
AST	0.061	0.530
ALP	0.045	0.642
γ-GT	0.109	0.262
IgM	−0.048	0.622
IgG	0.086	0.380
IgA	0.004	0.971
IgE	0.024	0.803
Child–Pugh score	0.187	0.540

RBC, red blood cell; WBC, white blood cell; PLT, platelet count; CRP, C-reactive protein; ALT, alanine aminotransferase; AST, aspartate aminotransferase; ALB, albumin; ALP, alkaline phosphatase; γ-GT, γ-glutamyl transpeptidase; TBil, total bilirubin; DBIL, direct bilirubin; IBIL, indirect bilirubin; IgM, immunoglobulin M; IgG, immunoglobulin G; IgA, immunoglobulin A; IgE, immunoglobulin E.

### Anxiety, depressive symptoms, and HRQoL

The prevalence of anxiety and depressive symptoms was 9.3 and 18.7%, respectively. As shown in Tables 4, 5, PBC patients with poor sleep tended to have higher GAD-7 and PHQ-9 scores, as well as lower PF, RP, and MH scores (all *p* < 0.05). The severity of sleep disturbance in PBC patients was significantly associated with the level of anxiety and depression (Figure 1).

**Table 4:** Results of the sleep state and other symptom assessment tool for patients with PBC.

	Good sleep (*n* = 45)			Poor sleep (*n* = 62)			
	Q1	Q2	Q3	Q1	Q2	Q3	*p*
GAD-7	0	0	2	2	5	8	*p*<0.001
PHQ-9	0	0	2	4	5.5	10	*p*<0.001
HT	0	50	50	25	50	75	0.902
PF	80	85	95	65	80	90	0.022
RP	75	100	100	0	75	100	0.007
BP	0	12	31	0	12	22	0.477
GH	42	47	52	45	52	57	0.230
VT	45	50	55	40	50	55	0.105
SF	62.5	62.5	62.5	50	62.5	62.5	0.109
RE	66.67	100	100	33.33	100	100	0.264
MH	52	60	68	48	56	60	0.002
F-VAS	3	5	7	2	5	6	0.286

Data were median (interquartile range), proportions, or simple frequencies as appropriate. The first quartile (Q1) is the value under which 25% of data points are found when they are arranged in increasing order. The second quartile (Q2, or the median) is the 50th percentile, meaning that 50% of the data fall below the second quartile. The third quartile (Q3) is the value under which 75% of data points are found when arranged in increasing order. GAD-7, Generalized Anxiety Disorder Scale; PHQ-9, Patient Health Questionnaire 9; HT, health transition; PF, physical functioning; RP, role physical; BP, bodily pain; GH, general health; VT, vitality; SF, social functioning; RE, role emotional; MH, mental health; F-VAS, Fatigue Visual Analog Scale.

**Table 5:** Correlation between sleep state and GAD-7, PHQ-9, SF-36, and F-VAS scores for patients with PBC.

	*r*	*p*
GAD-7	0.561	*p*<0.001
PHQ-9	0.652	*p*<0.001
HT	0.033	0.735
PF	−0.110	0.258
RP	−0.173	0.074
BP	−0.060	0.539
GH	0.133	0.174
VT	−0.175	0.072
SF	−0.106	0.279
RE	−0.032	0.744
MH	−0.343	*p*<0.001
VAS	0.022	0.821

GAD-7, Generalized Anxiety Disorder Scale; PHQ-9, Patient Health Questionnaire 9; HT, health transition; PF, physical functioning; RP, role physical; BP, bodily pain; GH, general health; VT, vitality; SF, social functioning; RE, role emotional; MH, mental health; F-VAS, Fatigue Visual Analog Scale.

### Diagnostic performance of the combination of PSQI, GAD-7, and RBC in assessing the impaired liver function (child–Pugh B–C) of PBC

**Figure 2:**
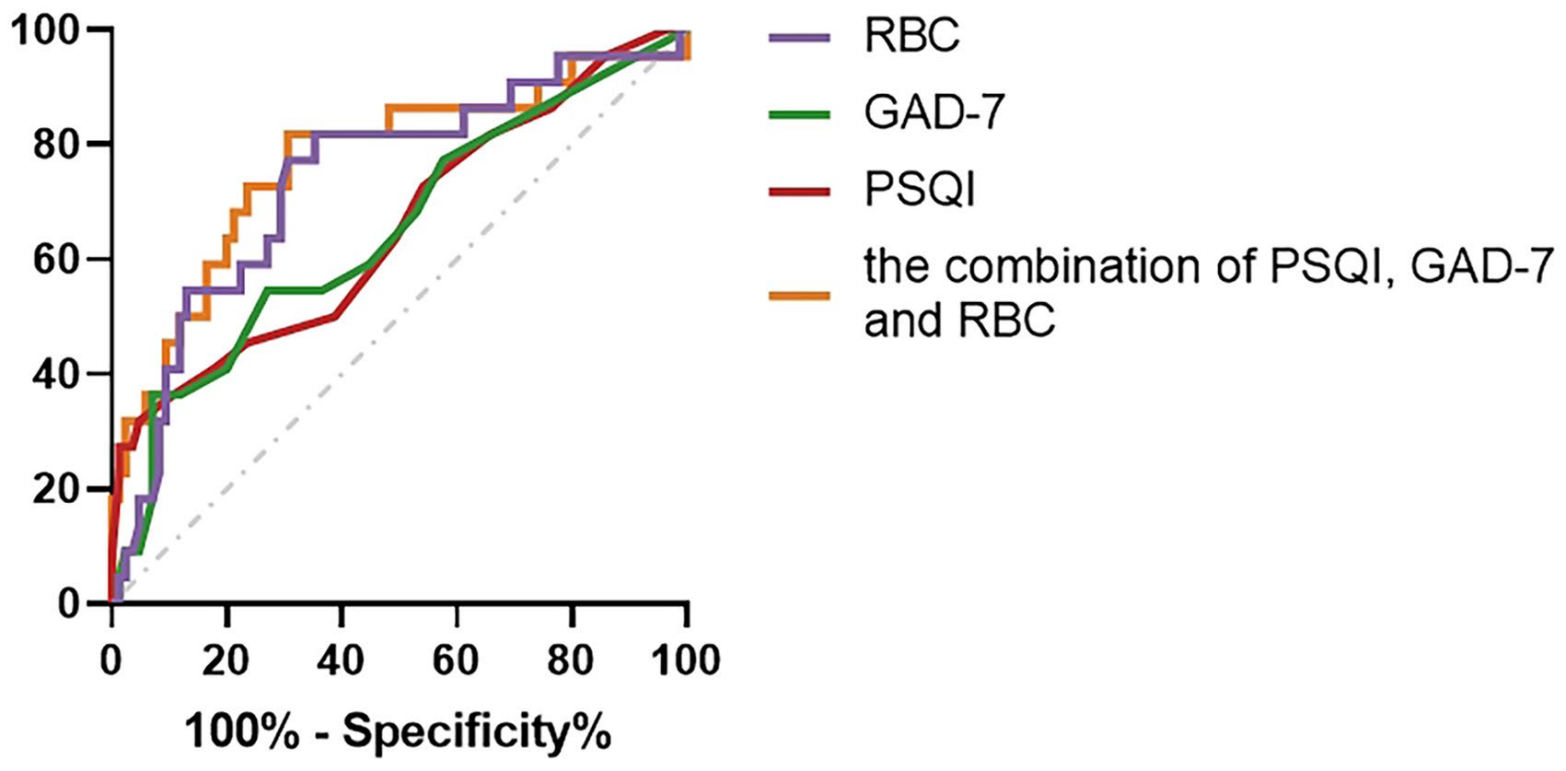
Compared with the combination of PSQI, GAD-7, and RBC, the diagnostic capabilities of the PSQI, GAD-7, and RBC values for assessing the impaired liver function (Child–Pugh B–C) of PBC.

Compared with the combination of PSQI, GAD-7, and RBC, the diagnostic capabilities of the PSQI, GAD-7, and RBC had fewer values for assessing the impaired liver function (Child–Pugh B–C) of PBC (Table 6; Figure 2).

**Table 6:** Diagnostic performance of the PSQI, GAD-7, RBC, and the combination of PSQI, GAD-7, and RBC in assessing the impaired liver function (Child–Pugh B–C) of PBC.

	Cut off	AUC	Sensitivity (%)	Specificity (%)	*p*
PSQI	11.5	0.654 (0.517 to 0.791)	31.8	95.3	0.026
GAD-7	8.5	0.649 (0.513 to 0.785)	36.4	92.9	0.032
RBC count(10^12^/L)	3.665	0.740 (0.617 to 0.862)	77.3	30.6	0.001
the combination of PSQI, GAD-7, and RBC count	0.193	0.771 (0.648 to 0.895)	81.8	30.6	*p*<0.001

PSQI, Pittsburgh Sleep Quality Index questionnaire; GAD-7, Generalized Anxiety Disorder Scale; RBC, red blood cell.

## Discussion

PBC is a chronic liver disease that can progress to cirrhosis, whose symptoms of depression and anxiety can interfere with sleep (18). As the most important sleep disturbance, the incidence of insomnia varies from 5.8 to 20% among the adult population in wider prospects (19). However, the incidence of PBC-related insomnia is much higher than that of primary insomnia, revealing that PBC has a unique pathogenesis (4–6).

There were copious findings that sleep disturbance appeared to be prevalent in PBC patients (20, 21). Basically, sleep disturbances had a negative efficaciousness to psychological, HRQoL, and biochemical markers (18, 22). Patients with chronic liver disease exhibited psychological impairments through extrahepatic pathways. The trajectory of chronic disease and the patient’s ability to cope with the disease are exceedingly determined by them, which reduces perceived HRQoL (23). The patient’s HRQoL can exert influence on the patient’s biochemical parameters in turn (9). It is imperative to analyze the interaction between this sleep disturbance and other psychological factors as well as serology so as to render the theoretical basis for ameliorating PBC patients with sleep disturbance.

A pivotal finding of the current investigation was that PBC patients with poor sleep quality had lower albumin levels. Meanwhile, the sleep disturbance was strongly correlated with the severity of anxiety, depression, and RBC count in PBC patients. Our study revealed that the combination of PSQI, GAD-7, and RBC was effective in assessing impaired liver function (Child–Pugh B–C) in PBC. Some aspects of HRQoL have been shown to be significantly correlated with age and albumin levels (6). Recent research suggested that sleep disturbance was closely associated with a high risk of malnutrition in patients with liver cirrhosis, and albumin was a crucial factor in the assessment of malnutrition (22). Several feasible mechanisms had been suggested that high malnutrition risk resulted in impaired sleep quality. At first, chronic inflammation had been widely demonstrated to be associated with sleep disturbances, and elevated inflammatory cytokines might modulate sleep behavior in patients with cirrhosis (24). On the other hand, liver diseases were orchestrated by a labyrinthine network of cytokine-mediated signaling pathways (25). Aberrant expression of inflammatory cytokines augmented the time of non-rapid eye movement (NREM) sleep, while peculiar antagonists of these cytokines alleviated the time of NREM sleep (26). In addition, the rampant and incessant inflammatory response also predisposed individuals to malnutrition by increasing muscle catabolism and resting energy expenditure (27). Finally, it had been proposed that cirrhosis had increased the risk of daytime melatonin levels and delayed melatonin peak onset during the night (28, 29). Circadian rhythms were synchronized by melatonin, a hormone secreted by the pineal gland in the dark, which was considerably higher at night than during the day (30). The disturbance of the circadian rhythm involved in melatonin increases the risk of PBC patients to have sleep disturbance. In this context, albumin and RBC levels were remarkably correlated with PBC patients’ sleep disturbance. Rising albumin and RBC levels in PBC patients might give assistance to ameliorate their sleep quality, and it is possible that this could further enhance HRQoL.

Examining the relationship between objective serological indicators and sleep disturbance might boost the augmentation of sleep through the rectification of objective conditions in the future. However, the study did not find dissimilarity in the anti-ENA antibody profiles of PBC patients with good and poor sleep quality. This indicated that the mechanism of sleep disturbance in PBC was not caused by autoantibodies.

There were several methods to measure the symptom burden and HRQoL in PBC patients, such as GAD-7, PHQ-9, PBC-40, and SF-36 (18). They had the dual character in gaging HRQoL of PBC patients (18). A European study illustrated that a large proportion of PBC patients had abnormal sleep, mainly manifested as daytime sleepiness (5). PBC patients had severely impaired HRQoL, which was substantially associated not only with fatigue, anxiety, and depression but also with gender, age, and body mass index (9, 31). The prevalence of sleep disturbances in this study was analogous to other studies, but the prevalence of anxiety and depression was lower than in other studies. This might be attributed to the different quantification methods. At the same time, sleep disturbance was profoundly connected to anxiety and depression in PBC patients in this study. Depression and anxiety in PBC might incorporate a multifactorial rationalization. The recent study hypothesized that the disruption of neurotransmitter synthesis and the modification in the brain structure seen on imaging were directly caused by the symptom burden of fatigue and itching (18). In fact, antidepressants and anxiolytics were indispensable to improve the psychological symptoms of PBC patients (32). A structural management to address these additional symptoms would represent a potential treatment for sleep disturbance.

HRQoL was significantly impaired in PBC patients, as measured by SF-36, regardless of sex or disease severity (10). Further research suggested that HRQoL was impaired in both Physical and social role (6). In this circumstance, there might be common characteristics of HRQoL injury in PBC patients. Sleep disturbance caused drastic impairment of the patient’s physiology and physiological functions, which might be related to complications such as bone disease, fat-soluble vitamin deficiency, hyperlipidemia, fatigue, and pruritus in PBC (18). Morning bright light therapy was beneficial to the amelioration of sleep disturbance, which had also been confirmed in some studies (20). Raising sleep disturbance in PBC patients provided potential value for boosting HRQoL. However, further large population follow-up and controlled studies are warranted.

In this study, differences in fatigue levels were not found among PBC patients with good and bad sleep. The interdependence between daytime sleepiness and fatigue in PBC patients had been observed in previous studies (5), and demographic background might be accountable for this corollary. Fatigue in PBC was independent of histopathological stage, degree of liver dysfunction, or autoantibody level (4, 33). The pathogenesis of fatigue in PBC remains poorly understood because of the multi-factor involved. Central nervous system abnormalities, autonomic nervous system abnormalities, abnormal levels of cytokines, fat, or progesterone metabolites, and peripheral muscle motor dysfunction might be interconnected with the pathogenesis of PBC fatigue (34).

The ultimate goal of this work was to ameliorate sleep and HRQoL in PBC patients. The effect of sleep quality on PBC patients’ lives was itself tricky. Although the presence of poor sleep quality showed a sizable association with HRQoL, patients with poor sleep quality rated their HRQoL as poor, which was associated with notable symptoms of social functioning. It demonstrated that sleep quality might eventually be altered by the extent to which they maintained social connections. This observation built on previous determinations revealed that coping strategies were predominant for perceptions of HRQoL in PBC (35). In effect, strengthening their social support might be a worthwhile way to help them augment PBC patients’ HRQoL and sleep in situations where medication was not appropriate. PBC patients also had plentiful selections of using natural remedies to diminish the burden of symptoms, such as yoga and meditation. They also had general health benefits, such as minimizing the risk of heart disease and stroke (36, 37).

However, the study had some unavoidable limitations: (i) This study was a single-center and cross-sectional study with a few patients. (ii) This study only incorporated the influence of some psychological variables on sleep quality. Psychological variables of self-efficacy, stress, and autonomic symptoms were not contemplated. (iii) This study used subjective rating scales to evaluate relevant data and lacked objective sleep and psychological assessment data, such as polysomnography or actigraphy. (iv) Multidimensional social environmental features, such as neighborhood deprivation and social cohesion, could also affect sleep quality, which was classified as a privacy issue, and it was difficult to obtain relevant information within local legal restrictions. Future studies should use objective tools to measure to obtain more accurate and rigorous psychological data on sleep. Multicenter, large sample, and follow-up cohort studies were needed to corroborate our conclusions, and more psychological variables should be included in these studies.

## Conclusion

Impaired HRQoL was remarkably associated with PBC patients with poor sleep. The combination of PSQI, GAD-7, and RBC was beneficial for preliminary screening impaired liver function. Based on the results of our research, ameliorating the depression and anxiety of patients and strengthening social support might be beneficial to the enhancement of patients’ sleep status. PBC patients with poor sleep had lower albumin levels, which might indicate a higher nutritional risk and a potential entry point for future treatment. However, it is necessary to further study the psycho-neuro-immunological pathway between PBC and sleep.

## Data Availability

The datasets presented in this article are not readily available because the data are not publicly available due to them containing information that could compromise research participant privacy. Requests to access the datasets should be directed to BL, binliu72314@163.com.
